# Determination of Albumin, Glucose, and Creatinine Employing a Single Sequential Injection Lab-at-Valve with Mono-Segmented Flow System Enabling In-Line Dilution, In-Line Single-Standard Calibration, and In-Line Standard Addition

**DOI:** 10.3390/molecules25071666

**Published:** 2020-04-04

**Authors:** Kanokwan Kiwfo, Wasin Wongwilai, Tadao Sakai, Norio Teshima, Kate Grudpan

**Affiliations:** 1 Center of Excellence for Innovation in Analytical Science and Technology, Chiang Mai University, Chiang Mai 50200, Thailand; k.kanokwan11@gmail.com (K.K.); wwongwilai@gmail.com (W.W.); 2 Department of Chemistry and Graduate programs in Chemistry, Faculty of Science, Chiang Mai University, Chiang Mai 50200, Thailand; 3 Department of Applied Chemistry, Aichi Institute of Technology, 1247 Yachigusa, Yakusa-cho, Toyota 470-0392, Japan; tadsakai@octn.jp (T.S.); teshima@aitech.ac.jp (N.T.); 4 Science and Technology Research Institute, Chiang Mai University, Chiang Mai 50200, Thailand

**Keywords:** SI-LAV, mono-segmented flow, in-line dilution, in-line single-standard calibration, in-line standard addition, albumin, glucose, creatinine

## Abstract

A mono-segmented sequential injection lab-at-valve (SI-LAV) system for the determination of albumin, glucose, and creatinine, three key biomarkers in diabetes screening and diagnosis, was developed as a single system for multi-analyte analysis. The mono-segmentation technique was employed for in-line dilution, in-line single-standard calibration, and in-line standard addition. This made adjustments to the sample preparation step easy unlike the batch-wise method. The results showed that the system could be used for both fast reaction (albumin) and slow reaction (glucose with enzymatic reaction and creatinine). In the case of slow reaction, the analysis time could be shortened by using the reaction rate obtained with the SI-LAV system. This proposed system is for cost-effective and downscaling analysis, which would be applicable for small hospitals and clinics in remote places with a small number of samples but relatively fast screening would be needed.

## 1. Introduction

The determination of biomarkers in clinical samples is essential for screening for diagnosis and/or medical treatment for many diseases. Albumin, glucose, and creatinine are, for example, the key biomarkers for diabetes mellitus. In a large hospital, the three biomarkers are usually determined by using an automatic analyzer, so as to serve a large number of the clinical samples. In some, each of the three biomarkers may be operated by using an individual analyzer. However, in some small hospitals, a few samples may be encountered. In the latter cases, as the hospitals may be in remote places, a simple, cost-effective instrument may serve the requirement for clinical analysis of the three biomarkers, but the determination of the three biomarkers should be carried out in a short analysis period.

Recently, the mono-segmentation method has been employed in various flow-based techniques such as simultaneous multiple injection, multi-commutated flow system, micro-titration, and sequential injection. The mono-segmentation method involves creating a stack zone between air segments for eliminating dispersion and dilution effects from the carrier stream. It has been used in corporation with various analytical techniques, such as the electrochemical method, spectrophotometric method, and titration method [1,2,3,4,5,6,7,8,9,10,11,12,13], with different tasks in the chemical analysis steps from sample preparation to detection via in-line sample dilution, in-line single-standard calibration, and in-line standard addition, as summarized in Table 1. Although there have been a number of reports regarding mono-segmentation, most of the works engaged the development in applying mono-segmentation for one or two of the three tasks (in-line sample dilution, in-line single-standard calibration, and in-line standard addition). There was only one report using mono-segmentation for the three tasks together by employing only a one-instrument setup for multi-analyte determinations. That work was with electrochemical techniques [8]. It is of interest to apply mono-segmentation in a one-instrument setup for the three tasks in steps of chemical analysis for assays of albumin, glucose, and creatinine for some specific aims.

Sequential injection analysis with lab-at-valve (SI-LAV) has been developed in order to offer a simple, cost-effective, alternative system for flow-based analysis. An additional component was attached to a multi-position selection valve, allowing various chemical reactions to be manipulated and monitored. With respect to the lab-on-valve (LOV) technique, the modification in LAV utilizes a device and tool available in laboratory, with no extra mechanical work required [14].

In this work, a simple, cost-effective SI-LAV system with mono-segmentation operation was proposed for downscaling chemical analysis, with the aim of developing a fully automated single system for the assays of the three biomarkers (albumin, glucose, and creatinine) for screening purposes in a small hospital or a clinic. With mono-segmentation, three operations were adopted from sample preparation to detection steps as follows: In-line sample dilution, in-line single-standard calibration, and in-line standard addition.

Chemical reactions used in the SI-LAV system involve the ion association of protein with tetra-bromophenolphthalein ethyl ester (TBPE) for albumin determination [15,16], an enzymatic reaction with p-anisidine as chromogenic reagent for glucose determination [16], and Jaffé reaction for creatinine determination [17,18,19].

## 2. Results and Discussion

### 2.1. Determination of Albumin

The detection reaction for albumin determination is based on the ion association of protein with tetra-bromophenolphthalein ethyl ester (TBPE) in the presence of Triton X-100 at pH 3.2 to form a blue product [15,16]. The albumin reaction was used as a model study of fast reaction.

In the sample preparation step, the mono-segmented technique was employed for in-line sample dilution (Table S1 and Figure S1A). The sample could be diluted 2–140-fold to achieve a concentration suitable for the detection range. Next, the mono-segmented technique was applied for in-line single-standard calibration (Figure S1B). In between air segments, the mono-segment of 200 µL was created. Each 100-µL mono-segment contained a sequence of R1: Albumin reagent (5.0 × 10^−5^ mol·L^–1^ TBPE, 0.02% Triton X-100, 0.04 mol·L^–1^ acetate buffer pH 3.2), W: deionized (DI) water, and SD: standard solution (human serum albumin (HSA)) at volumes of 65, 35 − Y and Y µL, respectively. Alternatively, the mono-segmented technique could be used in in-line standard addition (Figure S1C). A mono-segment of 200 µL was created, containing a sequence of R1, W, SD, R1, W, and S: sample at volumes of 65, 35 − X, X, 65, 35 − Y, and Y µL, respectively. During in-line standard addition, the dilution factor could be increased by aspirating the predilution sample from the port number 2 and adjusting the diluent volume accordingly. This flexible process made it more convenient for multi-dilution. In the previous study, it was suggested that the diluted sample could reduce the interference by at least 20-fold [15]. 

**Figure 1:**
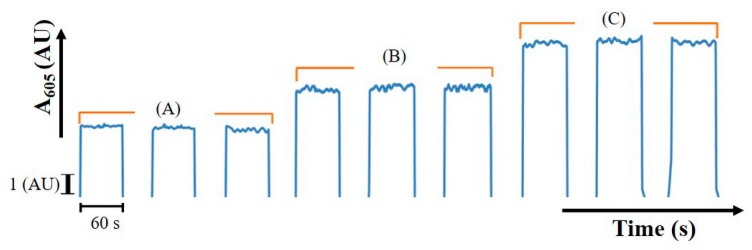
Analytical signal obtained from albumin content in 200 µL (**A**) 0, (**B**) 2.1, (**C**) 3.5 µg human serum albumin (HSA).

Due to the fast reaction rate of albumin with TBPE, the reaction could approach the steady state in a short time. The absorbance at 605 nm was monitored. It was found that the absorbance of product (A_605_) was proportional to albumin content, as shown in Figure 1. The linear range was up to 3.5 µg HSA in 200 µL, with a calibration equation of absorbance = 0.0558 (HSA (µg) in 200 µL) + 0.0491, r² = 0.992, limit of detection (LOD) (3σ) [20] = 0.4 µg in 200 µL. From the µg amount obtained in 200 µL, the concentration (mg/dL) of albumin in the original can be evaluated. 

### 2.2. Determination of Glucose

The detection reaction for glucose determination involves glucose oxidase (GOD) promoting the oxidation of d-(+)-glucose. Glucono lactone and hydrogen peroxide are produced. The hydrogen peroxide obtained from the oxidation of glucose oxidizes p-anisidine to a red color compound in the presence of iron (II) used as a catalyst [16].

For in-line sample dilution (Table S2 and Figure S2A), the mono-segmented technique for a dilution of glucose could flexibly be carried out for 2–160 folds.

For in-line single-standard calibration (Figure S2B), the mono-segment of 200 µL was created, similar to the albumin assay but with the enzyme solution aspirated separately from other reagents to avoid enzyme denaturation. It consisted of repeated sequences of R2: Glucose reagent (0.04 mol·L^–1^ p-anisidine, 0.002 mol·L^–1^ iron (II) in 0.002 mol·L^–1^ H_2_SO_4_, 0.1 mol·L^–1^ acetate buffer, pH 4.5), R3: Glucose oxidase, W: DI water, and SD: standard glucose at volumes of 50, 10, 40 - Y, and Y µL, respectively.

**Figure 2:**
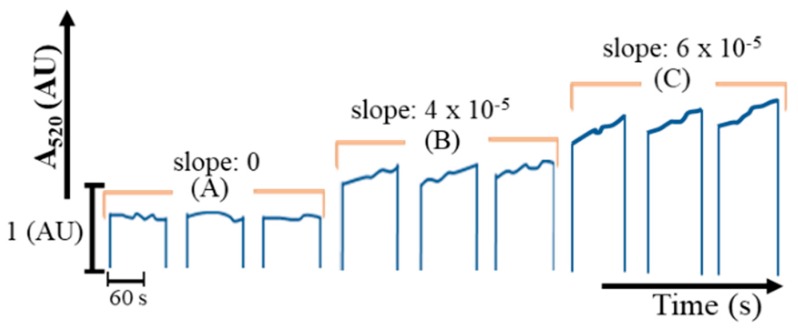
Analytical signal obtained from glucose content in 200 µL. (**A**) 0, (**B**) 2.4, and (**C**) 4 µg.

In the case of in-line standard addition (Figure S2C), the mono-segment of 200 µL was created with a sequence of R2, R3, W, SD, R2, R3, W, and S, at volumes of 50, 10, 40 - Y, Y, 50, 10, 40 – X, and X µL, respectively. The dilution factor could be increased by adjusting the prediluted sample volume for suitable concentration, similar to the albumin system. Absorbance at 520 nm in the reaction chamber was monitored by using stop-flow mode. This showed that absorbance increased with time (Figure 2).

Unlike the reaction of albumin, the reaction of glucose could be monitored using SI-LAV in a presteady state due to its relatively slow reaction rate. The reaction rate was found to be proportional to the concentration of glucose, as it complied with the pseudo first-order reaction, as previously reported [21]. The reaction rate was calculated using a slope of absorbance at 520 nm recorded at reaction time 30–120 s in the presteady state. The increase in the reaction rate was proportional to glucose concentration, which is directly correlated to glucose content. A linear range was obtained up to 4 µg glucose in 200 µL, with a calibration equation of reaction rate = 1.52 × 10^–5^ (glucose (µg) in 200 µL) + 1.00 × 10^–6^, r² = 0.986, LOD (3σ) [20] = 1.5 µg in 200 µL. From the µg amount obtained in 200 µL, the concentration (mg/dL) of glucose in the original can be evaluated.

In this study, the glucose oxidase in solution form was used for glucose determination, since it could exhibit stable activity across batch-wise preparation. It should be noted that the enzyme solution was readily homogenously mixed with the substrate, which helped shorten total analysis time by at least 10 times compared to the previous work using an enzyme-immobilized bead column [15]. Also, it was suggested that exploits of the standard addition method and reaction rate in calibration could reduce color interference, especially for urine samples.

### 2.3. Determination of Creatinine

The detection reaction for creatinine determination is based on the Jaffé reaction. Creatinine reacts with picrate in an alkaline medium to form a red-orange product [17,18,19].

First, the mono-segmented technique was applied for in-line creatinine sample dilution in the range of 2–100 folds (Table S3 and Figure S3A). It was reported previously that the creatinine sample had to be diluted by at least 80 fold in order to minimize the interference effect [19].

**Figure 3:**
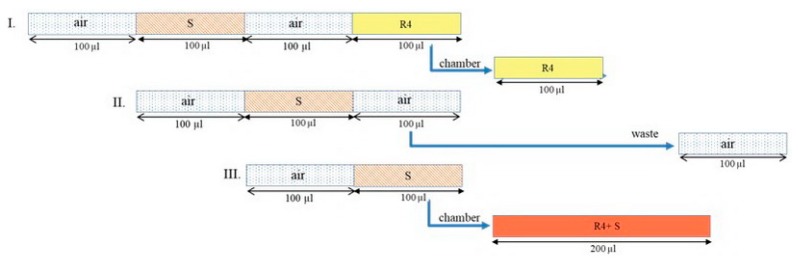
A sequence profile for creatinine determination (R4: Mixed reagent for creatinine determination (R4: 0.025 mol·L^–1^ sodium picrate, 0.75% NaOH, 0.03 mol·L^–1^ KH_2_PO_4_), S: Pretreated creatinine solution).

In the reaction and detection step (Table S4), the mono-segmented technique was applied in either in-line single-standard calibration or in-line standard addition. Unlike albumin and glucose assays, two mono-segments were created, as shown in Figure 3. First, the 100-µL mono-segment containing creatinine reagent was aspirated to into chamber (port number 10), followed by the dispensing of a 100-µL mono-segment of either creatinine standard or sample.

For in-line single-standard calibration, from above, another 100-µL mono-segment contained creatinine standard and DI water at volumes of X and 100 − X µL, respectively (Figure S3B). In the case of standard addition, another 100-µL mono-segment contained a pretreated sample (S), creatinine standard, and DI water (Figure S3C). The desired concentration could be achieved by adjusting the volume ratio. These mono-segments were aspirated into the LAV chamber successively, as shown in Figure 3.

**Figure 4:**
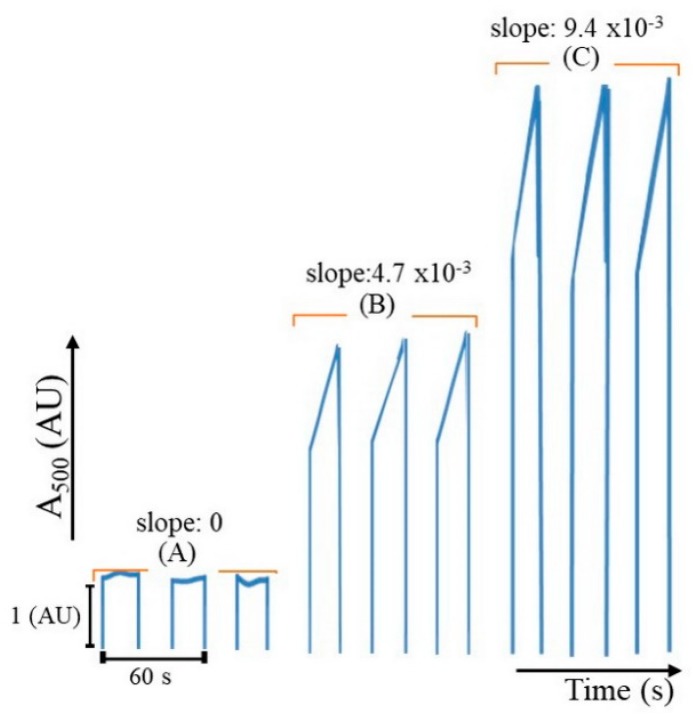
Analytical signal obtained from creatinine content in 200 µl (**A**) 0, (**B**) 10, and (**C**) 20 µg.

The two mono-segments were injected into the chamber (see steps E to H in Table S4). Using stop-flow mode allowed the reaction to be followed for kinetics (slope of the signal refers to a reaction rate). In Figure 4, the linear range was up to 20 µg creatinine in 200 µL, with a calibration equation of reaction rate = 4.71 × 10^–4^ (creatinine (µg) in 200 µL) − 2.00 × 10^–5^, r² = 0.996, LOD (3σ) [20] = 1.9 µg in 200 µL creatinine. From the µg amount obtained in 200 µL, the concentration (mg/dL) of creatinine in the original can be evaluated.

For glucose and creatinine determination, the results agreed with the previous study, showing that using kinetic data can reduce analysis time and could eliminate the effects of sample color and some interference in urine samples [19]. By using the mono-segmented SI-LAV system, the reaction rate of each reaction can be obtained. Those results from both reactions show that the use of the reaction rate to determine the concentration of analyte could be done in a shorter analytical time compared to those obtained at steady state. Therefore, the system could be useful, especially in the case of a slow reaction.

In all experiments, the in-line dilution using mono-segmentation allowed for the quick adjustment of the sample/standard volume to obtain the appropriate concentration within a linear range. If the first try with the dilution was unsuccessful, leading to the obtained signal being outside the linear range, the next attempt could be adjusted to be within the linear range. This could help shorten the sample preparation time significantly. In the detection step, the mono-segmentation technique provided a precise control of reaction mixture volume in a similar way to batch-wise practice, and thus the absolute concentration of the sample could be determined. This system could be applied for different analytical systems and further developed into a fully automated system for continuous multi-analyte analysis.

### 2.4. Preliminary Work in Application to a Real Sample

Using the developed SI-LAV system, preliminary work in the application to a real sample was performed for a urine sample, as shown in Table 2.

## 3. Experimental

### 3.1. Reagents and Chemicals

All chemicals used were of laboratory reagent grade. Deionized (DI) water, obtained from an Aquarius GSH-210 apparatus (Advantec, Tokyo, Japan), was used for preparing the solutions throughout.

For the albumin assay, human serum albumin (HSA) (Serologicals Proteins Inc., Kankakee, IL, USA), tetrabromophenolphthalein ethyl ester potassium salt (C_22_H_13_Br_4_KO_4_, Wako Pure Chemical Co., Osaka, Japan), and Triton X-100 (C_14_H_22_O(C_2_H_4_O)_n_ (*n* = 9–10), Fisher Scientific UK Ltd., Leicestershire, UK) were used.

For the glucose assay, d-(+)-glucose (C_6_H_12_O_6_, Sigma-Aldrich, St. Louis, MO, USA), p-anisidine (C_7_H_9_NO, Alfa Aesar, Lancashire, UK), iron(II) sulphate (FeSO_4_, Wako Pure Chemical Co., Japan), 98% sulfuric acid (H_2_SO_4_, QRëc, Auckland, New Zealand), glucose oxidase (E.C.1.1.3.4) from Aspergillus sp., 200U/mg, (Sigma-Aldrich, St. Louis, MO, USA), and 30% hydrogen peroxide (H_2_O_2_, BDH Prolabo, Lutterworth, UK) were used.

For creatinine assay, creatinine (C_4_H_7_N_3_O, Wako Pure Chemical Co., Osaka, Japan), potassium dihydrogen phosphate (KH_2_PO_4_, Wako Pure Chemical Co., Osaka, Japan), sodium picrate monohydrate (C_6_H_4_N_3_NaO_8_, Wako Pure Chemical Co., Osaka, Japan), and sodium hydroxide (NaOH, NACALAI TESQUE, INC., Kyoto, Japan) were used.

### 3.2. The Instrument Setup 

**Figure 5:**
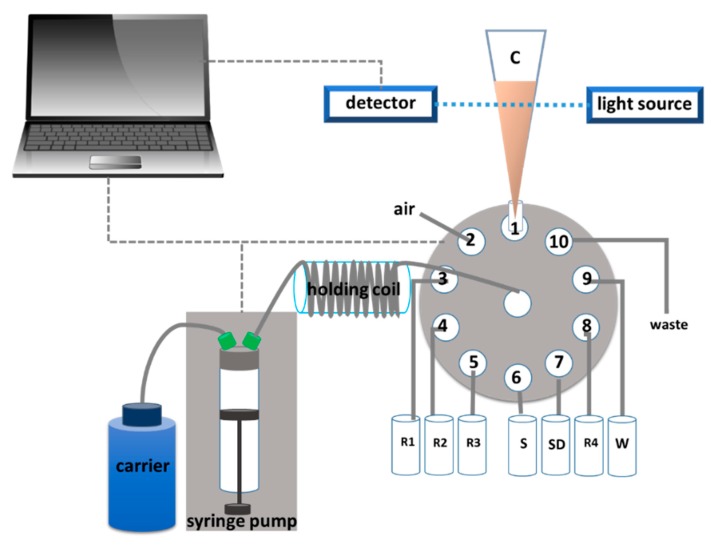
Schematic diagram of the sequential injection lab-at-valve (SI-LAV).

The SI-LAV system (Figure 5) was similar to that reported previously [22]. The system consisted of C: Mixing chamber and detection cell (micro pipette tip 1000 µL (Thermo Fisher, San Diego, CA, USA), light path length flow cell approximately 5 mm), R1: Albumin reagent (mixed solution of TBPE and Triton X-100 buffered at pH 3.2), R2: Glucose reagent I (mixed solution of p-anisidine and iron(II) buffered at pH 4.5), R3: Glucose reagent II (glucose oxidase), R4: Creatinine reagent (mixed solution of picric acid and sodium hydroxide), S: Sample, SD: Standard solution, W: DI Water, waste, syringe pump (2500 µL, FIA lab instruments, Seattle, WA, USA), 10-port selection valve (Valco Instruments Co. Inc., Houston, TX, USA), holding coil (PTFE Tubing, 0.5 mm i.d., 2.5 m long), spectrometer (USB4000, Ocean Optics, Largo, FL, USA), and fiber optics (P200-2UV/Vis Ocean Optics, Inc., Largo, FL, USA).

**Figure 6:**
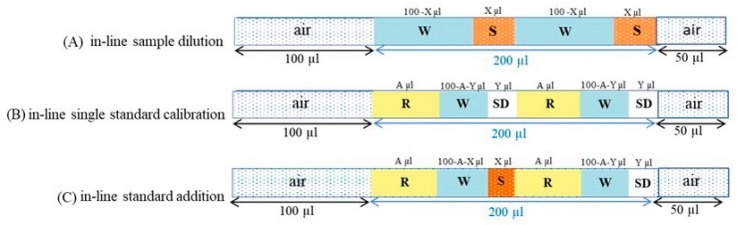
Operational sequences for (**A**) in-line sample dilution, (**B**) in-line single standard calibration, (**C**) in-line standard addition, W (DI water), S (sample), R (reagent), SD (standard solution).

In this work, the SI-LAV system was used for the determination of glucose, albumin, and creatinine. Sample and reagents were sequentially aspirated into an automatic control system. In-house software was used to control the pump and multi-port selection valve with various operational control sequences, as shown in Figure 6.

Within each mono-segment, the sample/standard solution could be split into a few small portions and sequentially aspirated in between diluent portions to increase the contract area, resulting in better mixing within the fixed volume of the mono-segmented zone. The mono-segmentation was employed in three sample preparation and detection steps as follows:

(1) For in-line dilution, the variable ratio of either sample (X µL) or standard solution (Y µL) and diluent (either 100 − X µL or 100 − Y µL) was aspirated twice within the fixed total volume of a 200-µL mono-segment between two air segments to yield the desired concentration, as illustrated in Figure 6(A). For dilution factors greater than 20, repeated in-line dilution was done by aspirating the first dilution segment to the port number 2 prior to detection.

(2) For in-line single-standard calibration, the fixed volume of reagent (A µL) combined with the variable ratio of standard solution (Y µL), and diluent (100 − A − Y µL) was aspirated twice within the fixed total volume of 200 µL mono-segment, as illustrated in Figure 6B.

(3) For in-line standard addition, the fixed volume of reagent (A µL) and sample (X µL) combined with the variable ratio of standard solution (Y µL) and diluent (200 − 2A − X − Y µL) was aspirated within the fixed total volume of 200 µL mono-segment between two air segments, as illustrated in Figure 6C.

## 4. Conclusions

The determination of albumin, glucose, and creatinine, which are the key biomarkers for diabetic screening diagnosis and medical treatment, employing a single sequential injection lab-at- valve with a mono-segmented flow system enabling in-line dilution, in-line single-standard calibration, and in-line standard addition, was proposed. Without needing to change the system configuration, the system enabled downscaling and automation approaches to be performed via in-line dilution, in-line single-standard, and in-line standard addition throughout the analysis process, which is convenient. With the system, a shorter analysis time via flow methodology can be obtained from the enzymatic reaction (for glucose) and a slow reaction (for creatinine). Development of the assay of the three analytes in one urine sample using the proposed system is in progress, with the aim of application in small hospitals and clinics with a small number of samples. This would reduce the time taken to transfer a sample to a larger laboratory.

## Figures and Tables

**Table 1:** Usages of mono-segmentation in sequential injection analysis.

No.	Analyte(s)/Sample (s)	Detection/Technique	Reagent	Role of Mono-Segmented			Ref.
				Sample Conditioning (Inline Dilution)	Inline Single Std. Calibration	Inline Std. Addition	
1	Fe(II) in pharmaceutical preparations, Cr(VI) in natural water and domestic waste water samples	solution handing for spectrophotometric determination (Fe(II) and Cr(VI))	KMnO_4_ for Fe(II) and diphenylcarbazide for Cr(VI)			X	[1]
2	Fe(II) in anti-anemic medicine	spectrophotometric determination of Fe(II)	1,10-phenanthroline	X	X	X	[2]
3	sulfide in waters	spectrophotometric detection	Fe(III) and N,N-dimethyl-p-phenylene diamine hydrochloride		X	X	[3]
4	atrazine	voltammetric detection			X	X	[4]
5	picloram in natural waters	voltammetric detection			X	X	[5]
6	Mg, Ca in water sample	flame atomic absorption spectrometric detection			X	X	[6]
7	methyl parathion in water sample	voltammetric detection			X	X	[7]
8	Zn(II), Cd(II), Pb(II) and Cu(II) in water samples	voltammetric dectection		X	X	X	[8]
9	Al in water and beverage samples	tritrarion with spectrophotometric determination	sodium hydroxide as a titrant and phenolphthalein or thymolphthalein indicator		X		[9]
10	benzoic acid in a real beverage sample	amperometric detection	biosensor is based on the inhibition effect of benzoic acid on the biocatalytic activity of tyrosinase, polyphenol oxidase.	X	X	X	[10]
11	B in plants	spectrophotometric detection	azomethine-H		X		[11]
12	Se (IV) in raw Se-enriched yeast	spectrophotometric detection	o-pheneylenediamine		X	X	[12]
13	Al in water and beverage samples.	spectrophotometric detection	Eriochrome cyanine R		X	X	[13]

**Table 2:** The determination of albumin, glucose, and creatinine in a urine sample *.

Biomarker	Concentration (mg/dL)
Albumin	1.8
Glucose	not detectable **
creatinine	129

* A urine sample taken from a healthy female aged 31 years old; ** less than LOD as described earlier.

## Supplementary Materials

The following are available online, Table S1: Operational steps of the SI-LAV system for albumin determination, Figure S1: Sequence profile for albumin determination, Table S2: Operational steps of the SI-LAV system for glucose determination, Figure S2: A sequence profile for glucose determination, Table S3: Operational steps of the SI-LAV system for on-line sample pre-treatment for creatinine determination, Figure S3: Sequence profile for on-line sample pre-treatment creatinine solution.
