# Supplementary material for: Determination of Albumin, Glucose, and Creatinine Employing a Single Sequential Injection Lab-at-Valve with Mono-Segmented Flow System Enabling In-Line Dilution, In-Line Single-Standard Calibration, and In-Line Standard Addition
Source: Molecules. 2020 Apr 4;25(7):1666. doi: 10.3390/molecules25071666 (PMC7180603; doi:10.3390/molecules25071666)
Supplement: Supplementary file 1 [file molecules-25-01666-s001.pdf]

molecules

MDPI

Article

Determination of Albumin, Glucose and Creatinine Employing A Single Sequential Injection Lab-At-Valve with Mono-Segmented Flow System Enabling In-Line -Dilution, In-Line -Single Standard Calibration and In-Line–Standard Addition

Kanokwan Kiwfo [1,2,3], Wasin Wongwilai [1,4], Tadao Sakai [3], Norio Teshima [3], and Kate Grudpan [1,2,4*]

1 Center of Excellence for Innovation in Analytical Science and Technology, Chiang Mai University, Chiang Mai, 50200, Thailand
2 Department of Chemistry and Graduate programs in Chemistry, Faculty of Science, Chiang Mai University, Chiang Mai, 50200, Thailand
3 Department of Applied Chemistry, Aichi Institute of Technology, 1247 Yachigusa, Yakusa-cho, Toyota 470-0392, Japan
4 Science and Technology Research Institute, Chiang Mai University, Chiang Mai, 50200, Thailand
* Correspondence: kgrudpan@gmail.com; Tel.: +66-53941917

Supplementary Materials

Operation step of SIA-LAV and analytical signals

Table S1. Operational steps of the SI-LAV system for albumin determination.

step	description	pump valve position	selection valve position	volume (µl)	flow rate (µl/s)	remark
A	Aspirate DI water to syringe pump	In	-	1000	50	
B	Aspirate air to holding coil	out	1	100	10	
*C	Aspirate R1/W/S/ R1/W/S to holding coil	out	3/9/6/ 3/9/6	65/15/20/ 65/15/20	2/2/2/ 2/2/2/	
D	Aspirate air to holding coil	out	1	50	10	completed mono-segmented
E	Dispense reaction zone mixing chamber, hold reaction zone in	out	1	250	250	measured absorbance 605 nm for 60 sec
F	Clean system	out	10			

*step C: S would be replaced by SD for on-line single standard calibration or standard addition.

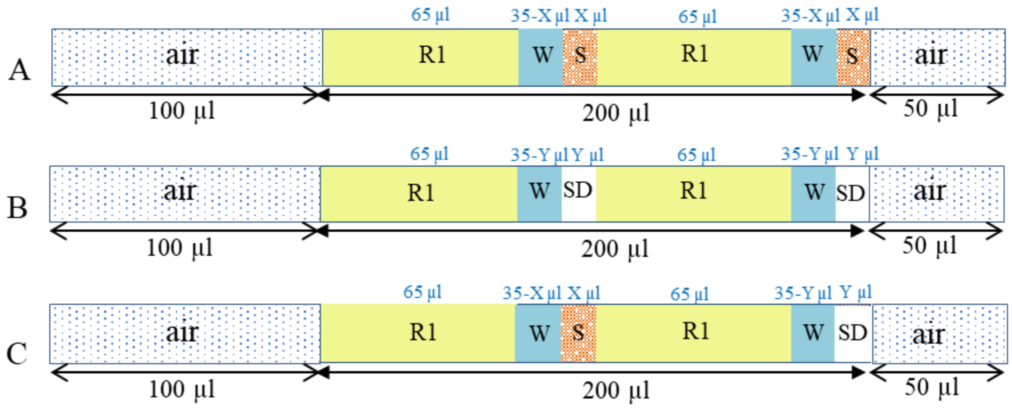

Figure S1. Sequence profile for albumin determination; (A) in-line sample dilution, (B) in-line single standard calibration; (C) in-line standard addition; W (DI water); S (sample); R1 (mixed reagent for albumin determination); SD (standard albumin solution).

Table S2. Operational steps of the SI-LAV system for glucose determination.

Step	Description	Pump valve position	Selection valve position	Volume (µl)	Flow rate (µl/S)	Remark
A	Aspirate DI water to syringe pump	in	-	1000	50	
B	Aspirate air to holding coil	out	1	100	10	
*C	Aspirate R2/ R3/W/S / R2/R3 W/S to holding coil	out	4/5/9/6/ 4/5/9/6	50/10/20/20 /50/10/22/18	2/2/2/2/ 2/2/2/2	
D	Aspirate air to holding coil	out	1	50	10	completed mono-segmented
E	Mixing of reaction zone by using flow reversal (n=2)	out		250	20	mixing reaction zone
F	Dispense reaction zone mixing chamber, hold reaction zone in chamber	out	1	250	250	monitored absorbance 520 nm, for 120 sec
G	Clean system	out	10	-	-	

*step C: S would be replaced by SD for on-line single standard calibration or standard addition.

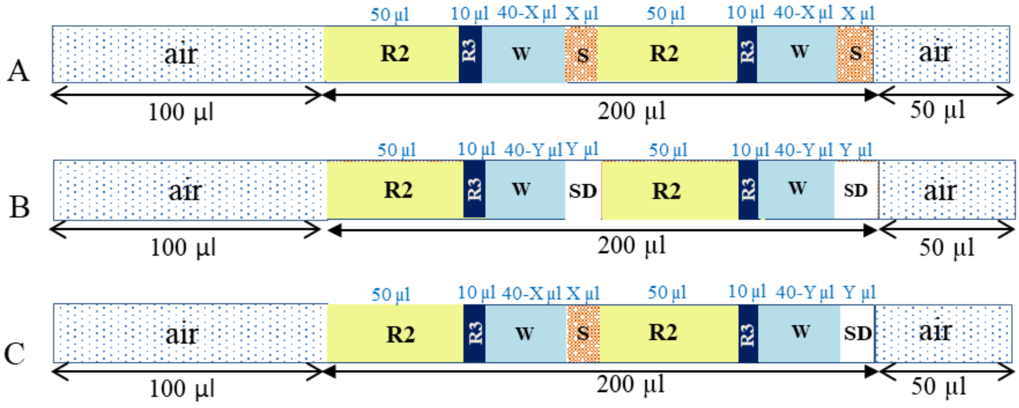

Figure S2. A sequence profile for glucose determination; (A) in-line sample dilution, (B) in-line single standard calibration; (C) in-line standard addition; W (DI water); S (sample); R2(mixed reagent for glucose determination); R3(glucose oxidase); SD (standard glucose solution).

Table S3. Operational steps of the SI-LAV system for on-line sample pre-treatment for creatinine determination.

Step	Description	Pump valve position	Selection valve position	Volume (µl)	Flow rate (µl/S)	Remark
A	Aspirate DI water to syringe pump	in	-	1000	50	
B	Aspirate air to holding coil	out	1	100	10	
*C	Aspirate W/S/W/S to holding coil	out	9/6/9/7	80/20/90/10	2/2/2/2	
D	Aspirate air to holding coil	out	1	50	10	completed mono-segmented
E	Flow reversal	out	1	250	20	
F	Aspirate standard added solution to holding	out	1	200	5	
G	Dispense standard added solution	out	2	200	5	
H	Clean system	out	10			

*step C: S would be replaced by SD for on-line single standard calibration or standard addition.

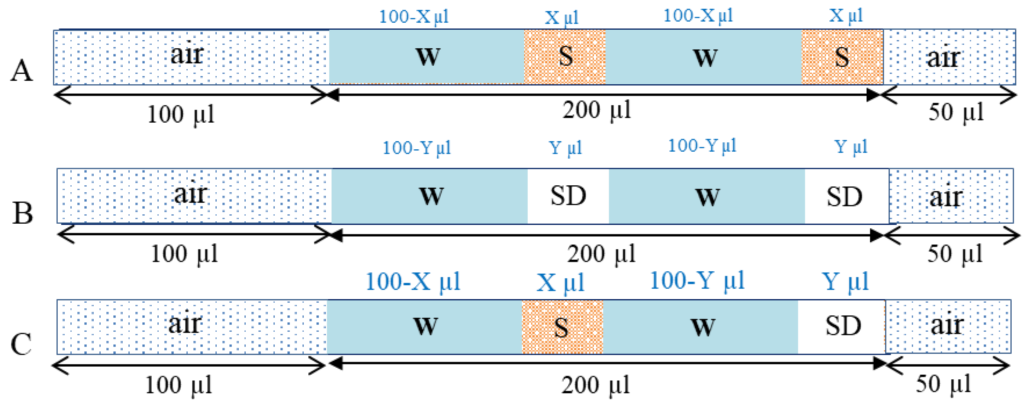

Figure S3. Sequence profile for on-line sample pre-treatment creatinine solution; (A) in-line sample dilution, (B) in-line single standard calibration; (C) in-line standard addition; W (DI water); S (sample); R2(mixed reagent for glucose determination); SD (standard creatinine solution).

Table S4. Operational steps of the SI-LAV system for creatinine determination.

step	description	pump valve position	selection valve position	volume (µl)	flow rate (µl/s)	remark
A	Aspirate DI water to syringe pump	in	-	1000	50	
B	Aspirate air to holding coil	out	1	100	10	
C	Aspirate pre-treated creatinine solution to holding coil	out	2	100	2	
D	Aspirate air to holding coil	out	1	50	10	
E	Aspirate R4 to holding coil	out	8	100	2	
F	Dispense R4 to mixing chamber	out	1	100	20	
G	Discard air	out	10	50	10	
H	Dispense standard added solution to mixing chamber, hold reaction zone in chamber	out	1	100	250	monitoring absorbance 500 nm for 20 sec
I	Clean system	out	10			
